# Supplementary material for: Health Benefits of Physical Activity Related to an Urban Riverside Regeneration
Source: Int J Environ Res Public Health. 2019 Feb 5;16(3):462. doi: 10.3390/ijerph16030462 (PMC6388232; doi:10.3390/ijerph16030462)
Supplement: Supplementary file 1 [file ijerph-16-00462-s001.pdf]

Supplementary Material

Health Benefits of Physical Activity Related to An Urban Riverside Regeneration

Cristina Vert, Mark Nieuwenhuijsen, Mireia Gascon, James Grellier, Lora E. Fleming, Mathew P. White and David Rojas-Rueda

Index

- **Table S1.** Questions included in the survey conducted by Barcelona local authorities in 2014–2015
- **Table S2.** Exposure-response function for each outcome
- **Table S3.** List of assumptions considered for the assessment of health and health-related economic benefits of the urban riverside regeneration project
- **Table S4.** Quartiles of basal levels of physical activity reported in Barcelona Health Survey
- **Table S5.** Direct health-care cost for morbidity outcomes in Spain
- **Table S6.** Descriptive analysis of the study population
- **Table S7.** Sensitivity analysis using the minimum visit duration to the Park reported by walking commuter users
- **Table S8.** Sensitivity analysis considering the minimum visit duration to the Park reported by cyclists >65 years old
- **Figure S1.** Exclusion criteria used to define the sample of survey respondents for analysis in this study
- **Figure S2.** Non-linear exposure-response function between physical activity in MET-hour/week and relative risks for all-cause mortality
- **Figure S3.** Formulae for the calculation of the relative risk used to estimate the health benefits for our study population.

Table 1. Questions included in the survey conducted by Barcelona local authorities in 2014-2015. These surveys were administered to Park users [1]. This is an adapted version of the original survey (in Catalan) and includes the key assumptions used for the model.

Questions *	Answers
1. Sex	o Men<br>o Women
2. Age	o _______ years old
3. Where do you live?	o Sant Adrià de Besòs<br>o Santa Coloma de Gramenet<br>o Badalona<br>o Montcada i Reixac<br>o Barcelona
4. Reason to come to the Besòs River:	o Walk (for pleasure)<br>o Sport–bicycle<br>o To walk to/from work<br>o To be healthy [a]<br>o Sport–run<br>o Others: _______(specify) [b]
5. Day when you usually come to the Park: (Multiple answer)	o Weekday (Monday–Friday)<br>o Saturday<br>o Sunday/holiday
6. Frequency [c]:	o More than 3 days/week<br>o 1 day/week<br>o 1 day/month<br>o Occasionally
7. Duration of the visit to the Besòs River [d]:	o Less than 1 hour<br>o 1–2 hours<br>o 2–4 hours<br>o >4 hours

* The original survey included more questions. However, in this table we only report questions that have been used for this study. [a,b] "To be healthy" and "Others" are not physical activity categories. Thus, users who answered any of these two options were excluded of the study sample. [c] In order to use these values in the analysis, we assumed that: "more than 3 days/week"="5.5 days/week" [considering that the maximum expected days/week would be 7, and the minimum expected days/week would be 4. Thus, the mean of these values would be: 5.5 = (7+4)/2]; "1 day/week"="1.0 day/week"; "1 day/month"="0.25 days/week" (0.25 = 1 day/ 4 weeks); "occasionally"="0.0 days/week". We assumed these values being as conservative as possible. [d]In order to use these values in the analysis, we assumed: "less than 1 hour"="0.5 hours/day"; "1–2 hours"="1.0 hours/day"; "2–4 hours"="2.0 hours/day"; ">4.0 hours"="4 hours/day". We assumed these values being as conservative as possible.

Table 2. Exposure-response function for each outcome [2–4].

Health outcome	Risk estimate [RR (95% IC)]	Exposure	Age group
All-cause mortality	0.810 (0.760, 0.850)	11 MET hours/week	≥18 years
IHD	0.909 (0.857, 0.964)	10 MET hours/week	≥18 years
Ischemic stroke	0.910 (0.831, 1.000)	10 MET hours/week	≥65 years *
Type 2 diabetes	0.980 (0.967, 0.996)	10 MET hours/week	≥18 years
Colon cancer	0.978 (0.940, 1.016)	10 MET hours/week	≥18 years
Breast cancer	0.987 (0.971, 1.003)	10 MET hours/week	≥18 years
Dementia	0.720 (0.600, 0.860)	33 MET hours/week	≥65 years *

- Exposure-response functions for ischemic stroke and dementia were available for subjects ≥65 years old. The study population was divided by age groups (18 to 64 years old, and ≥65 years old), with the aim of assigning appropriate age-specific incidence rates and exposure-response functions for each health outcome.

Table 3. List of assumptions considered for the assessment of health and health-related economic benefits of the urban riverside regeneration project.

**Assumption**	**Justification**
• The sample of survey respondents is representative of the study population. The survey was conducted by Barcelona local authorities and used in this study to estimate health and health-related economic benefits of the urban riverside regeneration project.	• Data and description of the procedure employed by local authorities to conduct the surveys was not available. However, this is official data which is being used by local authorities to assess the usability of the Besòs Riverside Park. To our knowledge, this is the only official data available at this moment.
• Scenario 1: 100% of the physical activity practised in the Besòs Riverside Park is new and related to the study intervention. Scenario 2: 50% of the physical activity practised in the Besòs riverside park is new and related to the study intervention (considered in the analysis), the other 50% was previously conducted somewhere else (e.g. on the beach, in a park, in the gym, etc) (not considered in the analysis).	• Data on the physical activity behaviour of the users of the Besòs Riverside Park before the intervention was not available. However, park infrastructure, including access to the riverbanks did not exist previously and the people could not use the area before the intervention completion.
• Base levels of physical activity of the study population are similar than those reported for the whole population of Barcelona [5,8]	• Data on the base levels of physical activity of the specific study population was not available. Nevertheless, this data was available at city level and it was expected to be similar among the Barcelona population and the study population.
• Survey data on frequency of the visits to the Park (Supplementary Material –Table S1), we assumed:<br>A) "more than 3 days/week"="5.5 days/week"<br>B) "1 day/month"="0.25 days/week"	• Based on a conservationist approach:<br>A) Maximum potential value = 7 days/week. Minimum potential value = 4 days/week. Thus, the mean of these values is: 5.5 = (7 + 4)/2.<br>B) 0.25 = 1 day/ 4 weeks.
• Survey data on duration of the visits to the Park (Supplementary Material –Table S1), we assumed:<br>C) "less than 1 hour"="0.5 hours/day"<br>D) "1–2 hours"="1.0 hours/day"<br>E) "2–4 hours"="2.0 hours/day"<br>F) ">4.0 hours"="4 hours/day"	• Based on a conservationist approach:<br>A) Maximum potential value=59 minutes. Minimum potential value=1 minute. Thus: 30 = (1 + 59)/2. And 30 minutes=0.5 hours.<br>B) Most conservative value.<br>C) Most conservative value.<br>D) Most conservative value.
• For Scenario 1, the estimated number of visits to the Park per day is 1 for all the Park users, except for the walking commuters, who need to go to and from work. Thus, for this group of users the number of visits per day is 2.	• Most conservative value.
• Non-linear exposure-response function between physical activity and health outcomes	• There is epidemiological evidence that suggest the exposure-response relationship between physical activity and health outcomes is non-linear [4].
• Data from 2014-2015 surveys (number of users, duration, frequency and type of physical activity) is assumed to be constant though the time	• Health benefits of physical activity do not emerge instantaneously and require regular practise. Data used for this study was collected 15 years after the completion of the riverside park, which means that the users who were using the infrastructure at this moment, may be users who have been using it for some time and that might continue using it in the future.

Table 4. Quartiles of basal levels of physical activity reported in the Barcelona Health Survey [5], a population-based randomized sample studying the health status of Barcelona residents. Levels of physical activity are reported in quartiles, Q1 being the lowest level of physical activity reported for the Barcelona population, and Q4 the highest level of physical activity.

Quartile	MET hour/week
Q1	0.4
Q2	8.5
Q3	22.5
Q4	42.4

MET: Metabolic equivalent of task.

Table 5. Direct health-care cost in euros for morbidity outcomes in Spain [6,7].

	Cost per case (euros)
IHD	1,123
Stroke	2,214
DM2	2,782
Colon cancer	3,031
Brest cancer	1,095
Dementia	5,830

Table 6. Descriptive analysis of the study population.

	**Total population: ≥18 years old** (N = 5,753)	**≥18 and ≤ 64 years old** (N = 2,848)	**≥65 years old** (N = 2,932)
**Age** [median (min-max)]	48 (18–85)	42 (18–64)	70 (65–85)
**Sex** (%)			
Men	65	61	78
Women	35	39	22
**Main activity conducted in the Besòs Riverside Park** (%)			
Walking for leisure	38	55	21
Cycling	49	19	78
Running	12	24	1
Walking for commuting	1	2	0

Table 7. Sensitivity analysis using the minimum visit duration to the Park (i.e. 30 min/day) reported by walking commuter users. Results for Scenario 1 and Scenario 2.

	Scenario 1			Scenario 2		
	**DALYs/year** (95% CI)	**Direct Costs (euros/year)** (95% CI)	**VSL (euros/year)** (95% CI)	**DALYs/year** (95% CI)	**Direct Costs (euros/year)** (95% CI)	**VSL (euros/year)** (95% CI)
Cycling	**−7.9** (−14.6; −2.4)	**−25,284** (−46,826; −9,108)	**−15,629,701** (−21,916,593; −11,401,939)	**−5.3** (−9.7; −1.7)	**−16,818** (−30,648; −6,149)	**−10,426,408** (−14,505,355; −7,651,506)
Walking for leisure	**−2.4** (−4.3; −0.7)	**−4,487** (−8,154; −1,608)	**−7,304,560** (−10,144,657; −5,367,509)	**−1.6** (−2.8; −0.5)	**−2,920** (−5,236; −1,059)	**−4,753,055** (−6,557,344; −3,510,218)
Running	**−0.8** (−1.4; −0.2)	**−146** (−264; −28)	**−460,256** (−643,971; −336,315)	**−0.5** (−0.9; −0.1)	**−99** (−178; −19)	**−305,284** (−423,907; −224,357)
Walking for commuting	**0.0** (−0.1; 0.0)	**−10** (−17; −2)	**−29,541** (−41,092; −21,682)	**0.0** (−0.1; 0.0)	**−6** (−11; −1)	**−19,300** (−26,663; −14,239)
TOTAL	**−11.1** (−20.4; −3.4)	**−29,926** (−55,262; −10,746)	**−23,453,984** (−32,746,312; −17,127,445)	**−7.4** (−13.5; −2.3)	**−19,843** (−36,074; −7,229)	**−15,504,047** (−21,513,268; −11,400,319)

DALYs: Disability Adjusted Life Years; VSL: value of statistical life.

Table 8. Sensitivity analysis considering the minimum visit duration to the Park (i.e. 30 min/day) reported by cyclists >65 years old. Results for Scenario 1 and Scenario 2.

	Scenario 1			Scenario 2		
	**DALYs/year** (95% CI)	**Direct Costs (euros/year)** (95% CI)	**VSL (euros/year)** (95% CI)	**DALYs/year** (95% CI)	**Direct Costs (euros/year)** (95% CI)	**VSL (euros/year)** (95% CI)
Cycling	**−6.3** (−11.4; −1.9)	**−16,256** (−29,574; −5,912)	**−15,629,701** (−21,916,593; −11,401,939)	**−4.2** (−7.5; −1.3)	**−10,591** (−19,006; −3,898)	**−10,426,408** (−14,505,355; −7,651,506)
Walking for leisure	**−2.4** (−4.3; −0.7)	**−4,487** (−8,154; −1,608)	**−7,304,560** (−10,144,657; −5,367,509)	**−1.6** (−2.8; −0.5)	**−2,920** (−5,236; −1,059)	**−4,753,055** (−6,557,344; −3,510,218)
Running	**−0.8** (−1.4; −0.2)	**−146** (−264; −28)	**−460,256** (−643,971; −336,315)	**−0.5** (−0.9; −0.1)	**−99** (−178; −19)	**−305,284** (−423,907; −224,357)
Walking for commuting	**−0.1** (−0.2; 0.0)	**−18** (−33; −1)	**−58,213** (−82,133; −16,786)	**−0.1** (−0.1; 0.0)	**−13** (−23; −2)	**−39,448** (−55,172; −28,834)
TOTAL	**−9.5** (−17.3; −2.8)	**−20,907** (−38,026; −7,550)	**−23,452,730** (−32,787,354; −17,122,548)	**−6.3** (−11.4; −1.9)	**−13,622** (−24,443; −4,979)	**−15,524,195** (−21,541,777; −11,414,915)

DALYs: Disability Adjusted Life Years; VSL: value of statistical life.

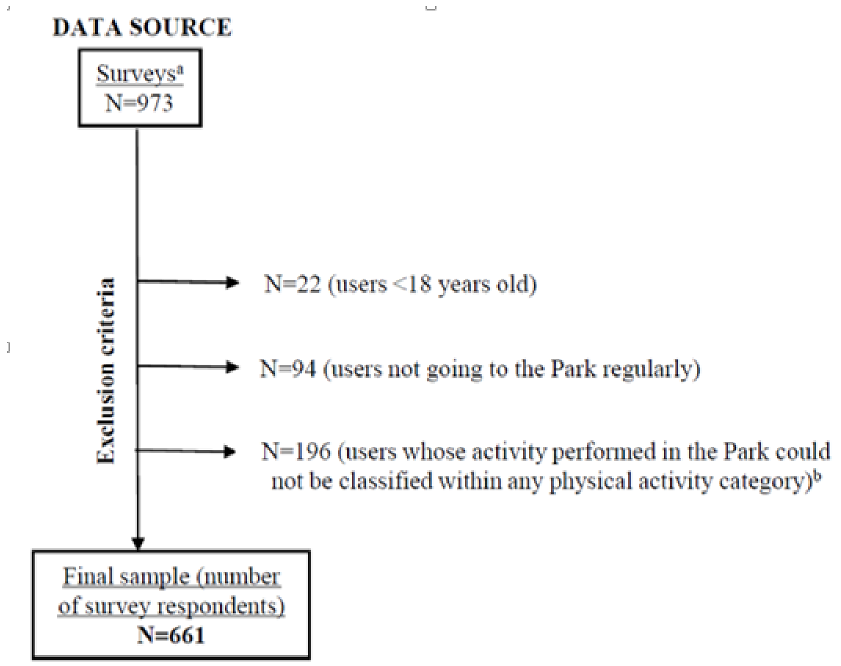

Figure 1. Exclusion criteria used to define the sample of survey respondents for analysis in this study. [a] Survey administered to Park users in 2014–2015 by Barcelona local authorities [1]. [b] Users who responded "to be healthy" or "others" as the "reason to come to the Besòs River" in the survey (see Supplementary Material – Table S1), were excluded of the sample of this study. This was because these activities could not be classified in a physical activity category according to the physical activity classification described by Ainsworth et al. 2011 [9]. This classification provides the energy cost of a wide variety of physical activities (e.g. dancing, walking, cycling, doing home activities like mopping or cleaning windows, etc.), which can be compared with other epidemiological studies providing data on self-reported physical activity.

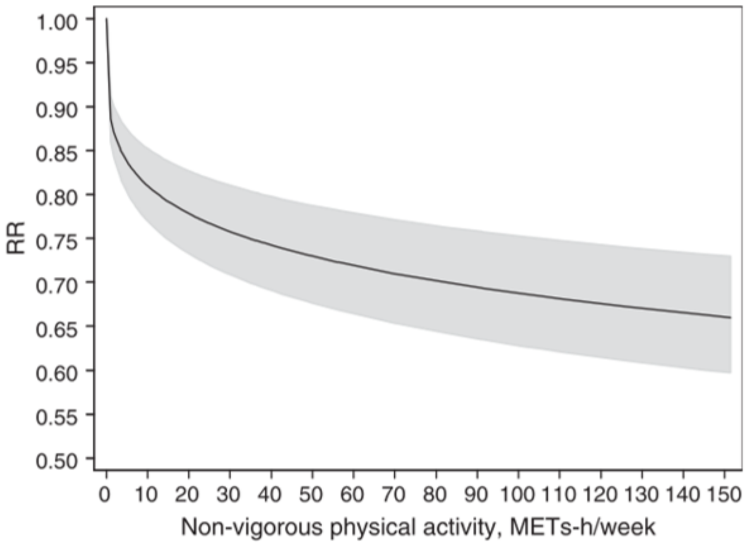

Figure S2. Non-linear exposure-response function between physical activity in METs hour/week and relative risks (RR) for all-cause mortality. Data obtained from a meta-analysis [4] including 22 studies. Shaded areas represent 95% confidence intervals.

$$RR\ for\ 1\ MET \frac{min}{week} = A^{(\frac{1}{B^{(y)}})}$$

$$RR\ basal = (RR\ for\ 1\ MET\ \frac{min}{week})^{C^{(y)}}$$

$$RR\ scenario = (RR\ for\ 1\ MET\ \frac{min}{week})^{D^{(y)}}$$

A = RR reference value. Risk estimate from exposure-response function obtained from meta-analysis [2–4]. See Supplementary Material – Table S2.
B = METs minutes/week reference value. Physical activity value from exposure-response function obtained from meta-analysis. See Supplementary Material – Table S2.
C = METs minutes/week basal value. Base levels of physical activity [5,8].
D = METs minutes/week basal + Scenario value. Base levels of physical activity [5,8] + estimated physical activity levels of the study population (Table 1).
Y = power transformation of 0.25 [4].

Figure 3. Formulae for the calculation of the relative risk (RR) used to estimate the health benefits for our study population.

References

1. Consorci Besòs. *Estudi de caracterització de l ' usuari del Parc fluvial del Besòs*; Sant Adrià de Besòs, Spain, 2015.
2. Hamer, M.; Chida, Y. Physical activity and risk of neurodegenerative disease: a systematic review of prospective evidence. *Psychol. Med.* **2009**, *39*, 3–11.
3. Kyu, H.H.; Bachman, V.F.; Alexander, L.T.; Mumford, J.E.; Afshin, A.; Estep, K.; Veerman, J.L.; Delwiche, K.; Iannarone, M.L.; Moyer, M.L.; et al. Physical activity and risk of breast cancer, colon cancer, diabetes, ischemic heart disease, and ischemic stroke events: systematic review and dose-response meta-analysis for the Global Burden of Disease Study 2013. *BMJ* **2016**, *354*, i3857.
4. Woodcock, J.; Franco, O.H.; Orsini, N.; Roberts, I. Non-vigorous physical activity and all-cause mortality: Systematic review and meta-analysis of cohort studies. *Int. J. Epidemiol.* **2011**, *40*, 121–138.
5. Bartoll, X.; Salvador, M.; Allué, N.; Borrell, C. *Enquesta de Salut de Barcelona 2011*; 2013; ISBN 9788498504651.
6. Ding, D.; Lawson, K.D.; Kolbe-Alexander, T.L.; Finkelstein, E. a.; Katzmarzyk, P.T.; van Mechelen, W.; Pratt, M. The economic burden of physical inactivity: a global analysis of major non-communicable diseases. *Lancet* **2016**, *388*, 1311–1324.
7. Parés-Badell, O.; Barbaglia, G.; Jerinic, P.; Gustavsson, A.; Salvador-Carulla, L.; Alonso, J. Cost of disorders of the brain in Spain. *PLoS One* **2014**, *9*.
8. Idescat Padró municipal d'habitants Available online: http://www.idescat.cat/pub/?id=pmh (accessed on 12 March 2018).
9. Ainsworth, B.E.; Haskell, W.L.; Herrmann, S.D.; Meckes, N.; Bassett, D.R.; Tudor-Locke, C.; Greer, J.L.; Vezina, J.; Whitt-Glover, M.C.; Leon, A.S. 2011 compendium of physical activities: A second update of codes and MET values. *Med. Sci. Sports Exerc.* **2011**, *43*, 1575–1581.
